# Myeloablative Chemotherapy with Autologous Stem Cell Transplant for Desmoplastic Small Round Cell Tumor

**DOI:** 10.1155/2015/269197

**Published:** 2015-04-07

**Authors:** Christopher J. Forlenza, Brian H. Kushner, Nancy Kernan, Farid Boulad, Heather Magnan, Leonard Wexler, Suzanne L. Wolden, Michael P. LaQuaglia, Shakeel Modak

**Affiliations:** ^1^Department of Pediatrics, Memorial Sloan-Kettering Cancer Center, 1275 York Avenue, New York, NY 10065, USA; ^2^Department of Radiation Oncology, Memorial Sloan-Kettering Cancer Center, 1275 York Avenue, New York, NY 10065, USA; ^3^Department of Surgery, Memorial Sloan-Kettering Cancer Center, 1275 York Avenue, New York, NY 10065, USA

## Abstract

Desmoplastic small round cell tumor (DSRCT), a rare, aggressive neoplasm, has a poor prognosis. In this prospective study, we evaluated the role of myeloablative chemotherapy, followed by autologous stem cell transplant in improving survival in DSRCT. After high-dose induction chemotherapy and surgery, 19 patients with chemoresponsive DSRCT underwent autologous stem cell transplant. Myeloablative chemotherapy consisted of carboplatin (400–700 mg/m^2^/day for 3 days) + thiotepa (300 mg/m^2^/day for 3 days) ± topotecan (2 mg/m^2^/day for 5 days). All patients were engrafted and there was no treatment-related mortality. Seventeen patients received radiotherapy to sites of prior or residual disease at a median of 12 weeks after transplant. Five-year event-free and overall survival were 11 ± 7% and 16 ± 8%, respectively. Two patients survive disease-free 16 and 19 years after transplant (both in complete remission before transplant). 14 patients had progression and died of disease at a median of 18 months following autologous transplant. These data do not justify the use of myeloablative chemotherapy with carboplatin plus thiotepa in patients with DSRCT. Alternative therapies should be considered for this aggressive neoplasm.

## 1. Introduction

First described as a distinct entity in 1989 [1], desmoplastic small round cell tumor (DSRCT) remains a relatively poorly understood neoplasm. It is mainly a disease of adolescent and young adult males, usually presenting with widespread intra-abdominal tumors not restricted to particular organs, but related to serosal surfaces [2]. Pleural [3] or paratesticular [4] involvement can also occur but is less common. Rare sites include orbit [5], bone [6], kidney [7], lung [8], ovary [9], and soft tissues of hand [10] and neck [11]. There is often disseminated disease at the time of diagnosis. Metastatic sites include lymph nodes, liver, and lung [12]. DSRCT has a characteristic histological appearance: nests of undifferentiated, moderately pleomorphic small round cells are surrounded by abundant desmoplastic stroma [2]. Epithelial, neural, and muscle markers are typically coexpressed on immunohistochemistry [13]. Besides the typical histological features, DSRCT is distinguished from other small round blue tumors by the presence of the t(11;22)(p13:q12) chromosomal translocation [14]. This translocation leads to the fusion of the EWS gene to the tumor suppressor gene WT1 [15]. The resultant chimeric protein acts as an aberrant transcription factor and is probably tumorigenic [16].

Despite the demonstrated chemosensitivity, optimal therapy for this rare disease remains to be determined, and prognosis is currently extremely poor. We previously reported on an aggressive multimodality therapeutic approach including high-dose, multiagent chemotherapy (the P6 protocol), surgery, and radiation therapy for DSRCT [17]. Tumors consistently responded to alkylator-based chemotherapy, although complete remissions were usually not obtained with chemotherapy alone. In order to exploit this demonstrated chemosensitivity and possibly improve survival in patients with DSRCT, we dose-intensified chemotherapy using autologous hematopoietic stem cells to reverse the associated myeloablation. Patients were treated with high-dose carboplatin and thiotepa taking advantage of the known responsiveness of DSRCT to alkylator-based therapy, dose-response behavior of these agents, and manageable extra medullary toxicities. Topotecan was later included for three patients, in an attempt to take advantage of possible potentiation of activity of alkylating agents, as well as favorable toxicity profile. We report on the results achieved with this strategy.

## 2. Patients and Methods

Patients with DSRCT treated at Memorial Sloan-Kettering Cancer Center (MSKCC) were given the option to consent to this study. All patients, except for two, were enrolled on an IRB-approved therapeutic protocol (ClinicalTrials.gov identifier NCT00002515) designed to evaluate myeloablative chemotherapy (MA) followed by autologous stem cell infusion (ASCT) in patients with rare high-risk solid tumors between 1993 and 2004. Patients that are not enrolled in study were treated as per protocol (patients 6, 17, Table 1) after obtaining written informed consent for protocol chemotherapy and ASCT. Records from the latter were accessed after obtaining permission from MSKCC Institutional Review Board. The diagnosis of DSRCT was established by histological evaluation of tumor specimens at MSKCC.

All patients had received induction chemotherapy followed by surgical resection with the objective of achieving remission prior to ASCT. Eligibility criteria for ASCT included: (A) demonstration of “chemosensitive” disease: patients needed to be in complete remission (CR) defined as no radiological evidence of disease or have ≥50% decrease in one measurable parameter attributable to prior chemotherapy without evidence of progressive disease by any other parameter, (B) availability of ≥2 × 10^6^ CD34+ autologous hematopoietic stem cells harvested peripherally via leukapheresis or ≥10^8^ nucleated cells/kg via bone marrow (BM) harvest and (C) adequate renal, hepatic, pulmonary, and cardiac function.

Planned MA for 16 patients was thiotepa 300 mg/m^2^ by 3-hour intravenous (IV) infusion on days −8, −7, and −6 (total, 900 mg/m^2^) and carboplatin by 4-hour IV infusion on days −5, −4, and −3. Carboplatin was dosed for an area under the curve (AUC) of 7 mg/mL/min using the Calvert formula with a maximum daily carboplatin dose of 700 mg/m^2^ [18]. Following protocol amendments, for a further three patients (patients 3, 14, and 19, Table 2), topotecan 2 mg/m^2^/day via 30 min IV infusion was added on days −8, −7, −6, −5, and -4.

ASCT was carried out on day 0 with autologous bone marrow (BM) or peripheral blood stem cells (PBSCs). Granulocyte colony-stimulating factor (G-CSF) 5-to-10 *µ*g/kg/day was started on day +1. Standard post-ASCT care was provided including the prophylactic use of antibiotics, parenteral nutrition, and blood product support. Toxicities were recorded according to the Children's Cancer Group Toxicity and Complications Criteria based on NCI common toxicity criteria.

Patients underwent the extent of disease evaluation approximately four to six weeks after ASCT with computed tomography (CT) or magnetic resonance imaging (MRI) of the primary site and potential sites of metastases. For patients with evaluable disease before ASCT, response was classified as follows: CR; good partial response: >90% decrease in all disease parameters; partial response (PR): >50% decrease in all disease parameters, mixed response: >50% decrease in ≥1 disease parameters but not in all; stable disease (SD) <50% decrease in all disease parameters; and progressive disease (PD): new lesions or >25% increase in any disease parameter. These criteria are in continuity with our previous report and were chosen due to the disseminated nature of DSRCT [17].

Planned therapy after hematopoietic recovery after ASCT included maximally tolerated radiotherapy to sites at high risk for progressive disease. Other therapies could be administered after radiotherapy at the discretion of the treating physician.

Survival curves were generated according to the Kaplan-Meier method and comparisons between groups performed using log-rank test using SPSS (IBM, Armonk, NY).

## 3. Results

### 3.1. Patient Demographics

Nineteen patients with DSRCT, (16 male, 3 female) with a median age at diagnosis of 18.5 years (range 10–42 years) were treated with ASCT. Patient characteristics before ASCT are presented in Table 1. Sixteen patients did not have relapse or PD prior to MA: eight were in first CR and eight had chemosensitive but persistent disease. Three patients were treated after first relapse: one was in second CR and two had persistent disease that was chemosensitive to salvage therapy prior to ASCT.

### 3.2. Prior Therapy

16 patients had received induction therapy with P6 protocol as previously described [17]. Briefly, this consisted of 4 cycles of high-dose cyclophosphamide, doxorubicin, and vincristine, followed by 3 cycles of ifosfamide and etoposide. One patient (#9) was initially thought to have neuroblastoma and received therapy with ifosfamide, carboplatin, and etoposide prior to correction of diagnosis to DSCRT whereupon he received P6 protocol. Another patient (#17) was initially diagnosed with testicular germ cell tumor and was observed without further therapy after initial surgical excision; he received P6 protocol at relapse. A third patient (#14) was treated with 6 cycles of high-dose chemotherapy as induction: three with high-dose cyclophosphamide plus doxorubicin and vincristine followed by three with high-dose cyclophosphamide plus topotecan and vincristine.

### 3.3. MA and ASCT

All patients received planned MA at a median of 9.1 (range 5.7–25.3) months from diagnosis (Table 1). Median carboplatin dose was 500 mg/m^2^/day. The source of autologous hematopoietic stem cells was PBSCs for 9, BM for 7, and combination of PB+BM for 3 patients in whom insufficient number of PBSCs were available. Median cell dose was 4.1 (range 2.1–17.5) × 10^6^ CD34+ cells/kg. All patients were engrafted (defined as an absolute neutrophil count >500/*µ*L) at a median of 11 ± 3 (range 6–23) days following ASCT. Median time to platelet recovery (defined as transfusion-independent platelet count >20,000/*µ*L) was 15 ± 15 (range 9–51) days (Table 2).

### 3.4. Acute Toxicities

There were no treatment-related mortalities. As expected, all patients experienced grade 4 myelosuppression. Toxicities are described in Table 2 and include grades 3 and 4 mucositis (*n* = 8 and *n* = 1, resp.), grade 3 diarrhea (*n* = 3), grades 3 and 4 hyperbilirubinemia (*n* = 4 and *n* = 2, resp.), grade 3 and grade 4 sepsis (*n* = 1 and *n* = 2, resp.), grade 3 and grade 4 hemorrhagic cystitis (*n* = 2 and *n* = 1 resp.), grade 3 vomiting (*n* = 3), and grade 3 hypertension (*n* = 1). One patient, with moderate hearing loss prior to ASCT progressed to a grade 4 hearing loss. Median time to discharge from hospital was 21 ± 6 (range 15–37) days, at which point all toxicities except for hearing loss had reverted to < grade 3.

### 3.5. Responses and Post-ASCT Therapy

Response was evaluated in 9/10 patients with measurable disease prior to ASCT. Seven had SD, one had a mixed response, and one had PD. 17 patients received 3000 cGy whole abdominopelvic radiotherapy (RT) with boosts to the tumor bed sites, as well as sites of bulk metastatic disease, at a median of 12 (range: 6 to 34) weeks following ASCT (Table 3). Two patients were treated with additional therapy following ASCT, one with anti-GD2 anti-idiotypic vaccine A1G4 (#14) and another with irinotecan followed by oral etoposide (#15); both patients died of PD.

### 3.6. Survival

**Figure 1:**
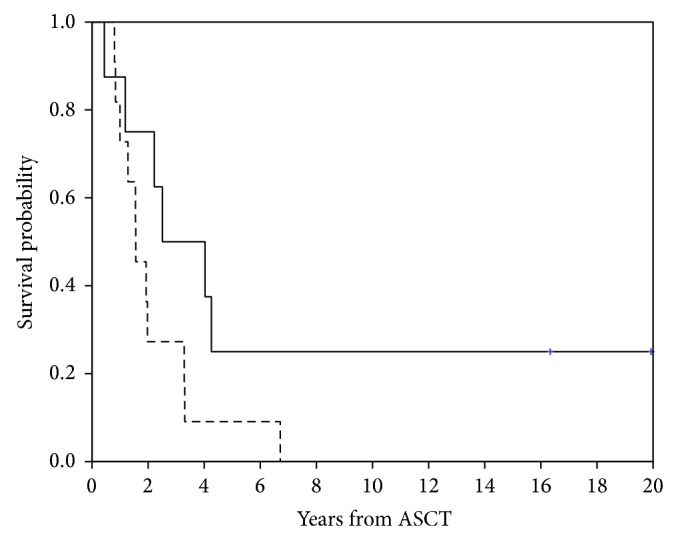
Overall survival probabilities of patients in first complete remission (—) prior to autologous stem cell transplant (ASCT) compared to all other patients (- - -).

Fifteen patients developed PD at a median of 12.8 (range 3.1–25.3) months after ASCT, one of whom (#1) died from complications of PD and sepsis 5 months later. Two patients (#13 and #17 Table 3) developed secondary acute myeloid leukemia (AML), 8 and 12 months after ASCT, and succumbed to this disease at 19 and 40 months after ASCT, respectively. Median time to death after relapse or development of AML was 12.4 months. Ten patients received various additional therapies following disease progression, which are detailed in Table 3. Two patients, neither of whom received any systemic therapy after ASCT, are currently alive without evidence of disease, 196 and 239 months after ASCT. Three-year event-free (EFS) and overall survival (OS) were 11 ± 7% and 26±10%, respectively, while 5-year EFS and OS were 11 ± 7% and 16 ± 8%, respectively. Median EFS and OS for patients in CR prior to ASCT were 13.7 ± 2.3 months and 30.1 ± 15.3 months from ASCT, respectively, while for those patients with measurable disease prior to ASCT EFS and OS were 9.1 ± 2.1 months and 18.7 ± 4.3 months, respectively. OS probability was higher for patients treated in the first CR compared to all other patients though this did not reach statistical significance (*p* = 0.07 for OS; *p* = 0.14 for EFS) in this small group of patients (Figure 1).

## 4. Discussion

The long-term prognosis for patients with DSRCT has essentially remained unchanged since the disease was first described as a distinct entity 25 years ago. Our group was the first to report responses to high-dose, alkylator-based chemotherapy, which has become the backbone of induction therapy for DSCRT [17]. However, responses were incomplete and aggressive debulking surgery to remove bulky residual disease is almost always necessary to try to achieve remission. In a retrospective analysis of a relatively large number of patients with DSRCT treated at MSKCC, multimodality therapy including high-dose alkylator chemotherapy in combination with aggressive surgical resection and radiotherapy resulted in improvement in intermediate-term (3 years) OS from 27% prior to the use of multimodal therapy to 55% after multimodal therapy was introduced [19]. Similar poor outcomes have been reported by other investigators treating patients with heterogeneous approaches without ASCT [20].

The rationale for the use of ASCT was to further escalate the doses of chemotherapy to overcome potential chemoresistance in disease that is regressing or has been rendered to a minimal state with induction chemotherapy and surgery in an effort to improve long-term outcomes. The toxicities of the chosen chemotherapeutic agents at high doses were primarily hematologic which could be overcome with ASCT. In our initial report, intermediate-term outcomes in patients treated with P6 induction followed by ASCT appeared to be favorable with 2 patients surviving disease-free 13 and 34 months from diagnosis [17]. However, as we now report on the data from a larger group of patients studied prospectively with longer followup, ASCT failed to improve outcomes, as patients treated with ASCT had a long-term survival of only 11%. Other investigators have made similar observations. A retrospective analysis by the Center for International Blood and Marrow Transplant Research of 36 patients with DSRCT treated in multiple centers with a variety of conditioning regimens revealed 3-year DFS of 23% [21]. Bertuzzi et al. reported on a cohort of 10 adult patients prospectively treated with high-dose melphalan plus mitoxantrone or thiotepa and found no improvements in overall survival [22]. Alternative approaches using sequential and multiple ASCTs administered earlier in the course of therapy were associated with equally poor outcomes [23]. Additional evidence of lack of effectiveness of ASCT was the poor response to the regimens used in our study with only a minor response noted in one patient. Moreover, the toxicity of ASCT was significant. Although there was no treatment-related mortality, the incidence of severe mucositis and hepatotoxicity was high. The apparently high incidence of secondary AML (2/19 patients) is likely a statistical anomaly related to the small number of patients undergoing ASCT.

Optimal “consolidation” therapy for patients whose disease burden has been significantly reduced remains to be determined. Based on our experience, ASCT appears to be ineffective in preventing relapse or progression for this group of patients even when post-ASCT whole abdominal radiotherapy is administered. Other options that could be considered for remission consolidation include conventional chemotherapy combinations such as irinotecan plus temozolomide [24, 25], vinorelbine, and low-dose cyclophosphamide [26]. Improvements in external beam radiotherapy using intensity-modulated approaches have significantly reduced radiation-related toxicity, but their efficacy in preventing relapse remains to be evaluated [27].

Directly targeting the peritoneal compartment, the site of relapse or progression in most patients with DSRCT might improve the outcome by eradicating minimal residual disease that is refractory or inaccessible to systemic chemotherapy. Such a strategy has shown to be of benefit for patients with ovarian carcinoma, another malignancy that involves the peritoneum [28] and for patients with malignant ascites [29]. Two such compartmental approaches are currently being studied in patients with DSRCT in early-phase trials. Intraperitoneal anti-B7H3 radioimmunotherapy with the radioiodinated monoclonal antibody 8H9 (NCT01099644) appears to be well tolerated with encouraging initial results in patients treated without measurable disease after surgery [30, 31]. Similar preliminary results have also been reported with hyperthermic intraperitoneal chemotherapy with cisplatin administered after tumor resection (NCT01277744) [32, 33]. However, the contribution of these interventions in preventing recurrence in patients who have been rendered into a state of minimal disease by surgery will be difficult to evaluate since objective responses cannot be measured.

Understanding the genomic aberrations and pathway abnormalities in DSRCT will be critical to the design of effective therapy. WT1 is a potential target for T-cell, natural killer cell or antibody-mediated immunotherapy. A number of downstream targets of the EWS-WT1 fusion protein such as insulin-like growth factor receptor [34] and platelet derived growth factor-A [35] are involved in growth factor signaling and could be inhibited with therapeutic benefit. Additionally, vascular endothelial growth factor- (VEGF-) A and VEGF receptor-2 are overexpressed in DSRCT [25] and the mTOR pathway is believed to be constitutively activated [36]. To exploit the vascular nature of DSRCT and this differential overexpression of VEGF-A and VEGF receptor-2, bevacizumab is being added to conventional chemotherapy (irinotecan/temozolomide + P6 protocol) in a trial for the upfront treatment of these patients (NCT01189643). Other agents being evaluated for activity in DSRCT include antitype-1 insulin-like growth factor receptor antibodies [37] and multiple tyrosine kinase inhibitors [38–41]. MTOR-inhibitors were found to downregulate the expression of the EWS/WT1 transcript and increase the Bax/BcL-xL ratio resulting in increased tumor cell death [42, 43]. Another potentially targetable aberration that appears unique to DSRCT is the equilibrative nucleoside transporter 4 (ENT4). ENT4 is a pH-dependent adenosine transporter that is directly activated by EWS/WT1 and highly expressed in primary tumors and cell lines, making it an attractive therapeutic target. It remains to be seen whether targeting any of these pathways, alone or in combination [44], has the potential to make a significant impact on patient outcomes.

Ours is the largest prospective study of myeloablative chemotherapy and ASCT in patients with DSRCT. While it supports the feasibility and tolerability of ASCT in patients with DSRCT after induction chemotherapy and surgery, it fails to demonstrate any clear benefit for ASCT in improving outcomes. A continued emphasis will need to be placed on developing and investigating novel approaches and therapies. The recent production of a panel of DSRCT cell lines might accelerate preclinical research in experimental therapeutics for this rare but lethal malignancy [45].

## Figures and Tables

**Table 1:** Patient characteristics before ASCT.

Pt#	Sex	Age at diagnosis (years)	Initial disease sites	Initial/induction therapy	Response to induction	Second line therapy	Diagnosis to ASCT (months)
First complete remission before ASCT							
1	F	11.9	Abdomen, pelvis	P6	CR	None	7.6
2	M	14.8	Abdomen, pelvis	P6	CR	None	7.6
3	M	15.0	Abdomen, pelvis	P6	CR	None	5.8
4	M	16.1	Abdomen, mediastinum	P6	CR	None	9.5
5	M	22.4	Abdomen, pelvis	P6	CR	None	5.7
6	M	24.1	Abdomen, pelvis	P6	CR	None	10.2
7	M	24.3	Abdomen, pelvis	P6	CR	None	6.8
8	F	47.7	Abdomen, mediastinum	P6	CR	None	10.1

Persistent but chemosensitive disease before ASCT							
9	M	10.0	Abdomen, pelvis	NB therapy followed by P6	PR	None	16.1
10	M	13.0	Abdomen, pelvis	P6	PR	None	7.8
11	F	13.6	Abdomen, pelvis, mediastinum, neck	P6	PR	None	9.8
12	M	16.3	Abdomen, pelvis	P6	PR	None	6.1
13	M	17.1	Abdomen, pelvis	P6	PR	None	9.1
14	M	20.7	Abdomen, pelvis, mediastinum	CAV/CTV	PR	None	7.9
15	M	31.7	Abdomen, pelvis, mediastinum	P6	PR	None	6.8
16	M	32.5	Abdomen, pelvis, neck	P6	PR	High-dose cyclophosphamide	10.7

Relapse before ASCT							
17	M	18.5	Testes	Surgery; observation	Not applicable	P6	25.3
18	M	22.4	Abdomen, mediastinum	P6	CR	High-dose cyclophosphamide; surgery	22.4
19	M	26.9	Abdomen, pelvis, neck	P6	PD	High-dose cyclophosphamide + topotecan	12.5

ASCT: autologous stem cell transplant; CAV: high-dose cyclophosphamide plus doxorubicin and vincristine; CR: complete remission; CTV: high-dose cyclophosphamide plus topotecan and vincristine; F: female; M: male; NB: neuroblastoma; P6: P6 protocol; PD: progressive disease; PR: partial remission.

**Table 2:** Myeloablative chemotherapy and autologous stem cell transplant: toxicities and response.

Pt#	Residual disease before ASCT	MA	Carboplatin dose (per m^2^)	Source of SC	Dose of SC (×10^6^ CD34+ cells/kg)	Time (days) to ANC >500	Time (days) to platelet count >20 K	Major acute nonhematological toxicities	Response ASCT
First complete remission before ASCT									
1	None	CT	700	PB + BM	3.0	13	51	Grade 3 hyperbilirubinemia, grade 3 hematuria	N/A
2	None	CT	500	BM	4.1	14	48	Grade 3 sepsis	N/A
3	None	CTT	700	PB	17.5	13	11	Grade 4 mucositis, grade 3 SGOT elevation	N/A
4	None	CT	700	PB + BM	3.4	13	34	None	N/A
5	None	CT	600	BM	5.6	9	28	Grade 3 diarrhea	N/A
6	None	CT	500	BM	3.2	13	26	Grade 3 hyperbilirubinemia, grade 3 mucositis	N/A
7	None	CT	450	PB	4.4	9	9	Grade 3 mucositis	N/A
8	None	CT	400	PB	UA	12	15	Grade 3 diarrhea, grade 3 vomiting	N/A

Persistent but chemosensitive disease before ASCT									
9	Abdomen	CT	500	BM	2.4	23	57	None	SD
10	Abdomen	CT	400	BM	2.6	11	16	Grade 3 mucositis	SD
11	Abdomen, pelvis, mediastinum	CT	425	PB	2.1	12	13	Grade 4 hyperbilirubinemia, grade 3 mucositis	Minor
12	Abdomen, mediastinum	CT	600	PB	7.2	9	14	Grade 4 sepsis	SD
13	Abdomen	CT	700	BM	3.1	14	41	Grade 4 hyperbilirubinemia	Lost to followup
14	Abdomen, mediastinum	CTT	500	PB	6.2	10	10	Grade 3 mucositis	SD
15	Abdomen, pelvis, mediastinum	CT	500	PB	4.1	6	13	Grade 3 hyperbilirubinemia, grade 3 vomiting	SD
16	Abdomen	CT	500	BM	2.4	10	11	Grade 3 hypertension, grade 3 mucositis	PD

Relapse before ASCT									
17	Mediastinum	CT	600	PB	5.1	9	15	Grade 3 vomiting	SD
18	None	CT	500	PB	5.0	10	13	Grade 3 mucositis, grade 3 diarrhea, grade 3 hematuria	N/A
19	Abdomen, axilla	CTT	600	PB + BM	UA	11	16	Grade 3 hyperbilirubinemia, grade 4 sepsis, grade 4 hearing loss, grade 4 hematuria, grade 3 mucositis	SD

ANC: absolute neutrophil count; ASCT: autologous stem cell transplant; BM: bone marrow; CT: carboplatin and thiotepa; CTT: carboplatin and thiotepa plus topotecan; FU: followup; MA: myeloablative chemotherapy; N/A: not applicable; PB: peripheral blood; PD: progressive disease; SC: stem cells; SD: stable disease; UA: details of dose unavailable.

**Table 3:** Therapy following ASCT and outcomes.

Pt#	Time to radiotherapy after ASCT (weeks)	Treatment at relapse	OS (months) after ASCT	EFS (months) after ASCT
First complete remission before ASCT				
1	No radiotherapy	None	5.3	1.4
2	12	N/A	239.1	239.1
3	10	Irinotecan/temozolomide; cyclophosphamide/vinorelbine; sunitinib; bevacizumab	48.4	13.7
4	15	Oral etoposide	30.1	13.2
5	12	Unknown	51.1	21.4
6	12	None	26.7	16.5
7	12	N/A	196.0	196.0
8	No radiotherapy	Paclitaxel, thiotepa	14.3	8.4

Persistent but chemosensitive disease before ASCT				
9	21	Vincristine, cyclophosphamide, dactinomycin	15.3	5.2
10	11	Palliative radiotherapy; oral etoposide	18.7	12.4
11	13	None	12.0	9.7
12	6	Exatecan	39.5	19.1
13	11	N/A	18.6	8.2 (developed secondary AML)
14	13	Temozolomide	23.8	16.4
15	13	Irinotecan, cisplatin; thalidomide; palliative radiotherapy	80.5	25.3
16	34	Vinorelbine	9.6	3.1

Relapse before ASCT				
17	12	N/A	39.7	12.1 (developed secondary AML)
18	12	Oral etoposide, vinorelbine, cisplatin, topotecan	23.2	8.7
19	14	None	10.1	7.1

AML: Acute myeloid leukemia; ASCT: autologous stem cell transplant; EFS: event-free survival; N/A: not applicable; OS: overall survival.
